# Ischemic intestinal stenosis complicated with portal and superior mesenteric vein thrombosis: Case reports and literature review

**DOI:** 10.1097/MD.0000000000041427

**Published:** 2025-02-07

**Authors:** Jie Li, Yuan Chang, Qian Ma, Jun Ke, Zhijian Ren, Zongliang Jia

**Affiliations:** a General Surgery Department, Xi’an International Medical Centre Hospital, Xi’an, Shaanxi, China; b General Surgery Department, Changzhou Second People’s Hospital, Nanjing Medical University, Jiangsu, China.

**Keywords:** case reports, computed tomography, intestinal infarction, ischemic intestinal stenosis, portal and superior mesenteric vein thrombosis

## Abstract

**Rationale:**

The main complication of the portal vein (PV) and superior mesenteric vein (SMV) thrombosis is intestinal infarction due to acute intestinal ischemia. However, in a small number of cases, chronic ischemia-induced bowel stenosis also occurs, and there are no published articles with a comprehensive literature review on this late complication. Therefore, we aimed to clarify the progression and clinical features of intestinal stenosis which is caused by thrombosis of the PV and SMV, and to share therapeutic experience.

**Patient concerns:**

We report 4 cases of ischemic intestinal stenosis occurring 4–8 weeks after successful treatment of PV and SMV thrombosis, and retrospectively analyzed the literature from 1990 to July 2024 for relevant cases to report.

**Diagnoses:**

All 4 patients were admitted to the hospital due to abdominal pain and were diagnosed with ischemic intestinal stenosis complicated with PV and SMV thrombosis through imaging studies.

**Interventions:**

The 4 patients received thrombolytic and anticoagulant therapy due to PV and SMV thrombosis. Subsequently, they underwent partial small bowel resection and double enterostomy because of the development of intestinal stenosis.

**Outcomes:**

Postoperative follow-up was conducted for the patients. Three patients underwent enterostomy reduction with a favorable prognosis, while 1 patient was lost to follow-up.

**Lessons:**

Patients with PV and SMV thrombosis should receive prompt anticoagulant or interventional therapy. In addition, closer follow-up should be carried out to ensure an earlier diagnosis of ischemic intestinal stenosis, leading to more timely treatment and a better prognosis.

## 1. Introduction

Thrombosis of the portal vein (PV) and superior mesenteric vein (SMV) is a relatively rare clinical condition of mesenteric ischemia associated with several risk factors, and also presents with nonspecific clinical manifestations, including abdominal pain, distension, diarrhea, or even asymptomatic cases.^[1]^ Advances in imaging technology and the increasing use of computed tomography (CT) in patients with acute abdomen have contributed to an improved diagnosis rate of rare conditions such as portal and SMV thrombosis.^[2]^

A major complication of PV and SMV thrombosis is intestinal infarction caused by acute intestinal ischemia, which usually requires emergency resection of the affected segment.^[3]^ However, in the presence of adequate collateral circulation and patency, acute intestinal ischemia may not occur, but chronic intestinal ischemia due to chronic venous outflow obstruction may lead to the development of intestinal stenosis, which is a rare complication of late PV and SMV thrombosis.^[1]^ This article reports 4 cases of intestinal stenosis due to PV and SMV thrombosis and provides a comprehensive literature review and analysis of this rare complication to elucidate the characteristics of this disease manifestation and to increase clinicians’ awareness of intestinal stenosis due to PV and SMV thrombosis.

## 2. Patients and methods

A retrospective study was conducted on the clinical data of 4 patients with intestinal stenosis caused by thrombosis of the portal and SMVs in our hospital from January 2023 to December 2024. All patients were informed and signed written informed consent. Considering the rarity of the complication of intestinal stenosis after PV and SMV thrombosis, we used PubMed, China National Knowledge Infrastructure, VIP China Science and Technology Journal Database, and Wanfang database to search for relevant cases published between 1990 and July 2024.

## 3. Case reports

### 3.1. Case 1

A 37-year-old female patient was admitted to our hospital for emergency treatment after complaining of abdominal pain and bloating. A contrast-enhanced CT scan conducted at a previous medical facility revealed diffuse thrombosis within the PV system. The patient had a documented 9-year history of ulcerative colitis with limited response to treatment. On physical examination, the patient had tenderness and rebound tenderness throughout the abdomen, accompanied by mild abdominal muscle tension. A pale red ascites was extracted through abdominal puncture. The laboratory findings revealed leukocytosis [count of 27.06 × 10^9^/L, with predominant neutrophilia (neutrophilic granulocyte percentage is 95.60%)], a raised C-reactive protein of 175.39 mg/L, elevated D-dimer level (16.84 mg/L), and moderate hypokalemia (K:2.6 mmol/L). Acute peritonitis was thought to be caused by extensive PV thrombosis resulting in obstruction of blood flow to the intestines. Although the possibility of bowel necrosis was considered, the patient refused emergency surgery and was instead treated with thrombolytic and anticoagulant therapy (30,000 units of urokinase twice a day was applied for 6 days). Following successful thrombolysis and anticoagulation, the patient experienced relief of abdominal symptoms and was prescribed ongoing anticoagulant therapy on discharge (fondaparinux 2.5 mg/day, subcutaneously).

**Figure 1.**
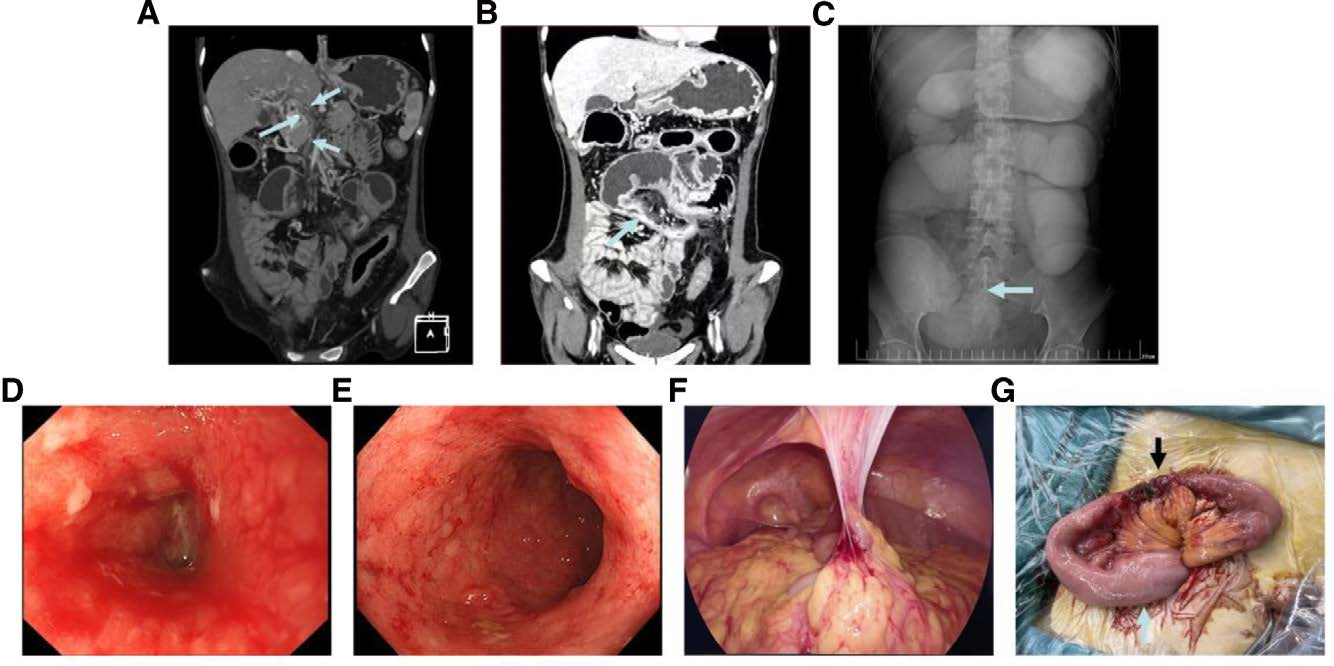
(A) Portal and superior mesenteric vein thrombosis (blue arrow). (B) A long segment of intestinal stenosis (blue arrow) with significant proximal bowel dilatation. (C) Digestive tract radiography revealed localized small bowel stenosis with a bird-beak sign (blue arrow). (D) Enteroscopy revealed circumferential jejunal stenosis with ulceration and obstruction. (E) Mucosal congestion and edema of the whole colon, loss of vascular texture and normal structure of the colon. (F) A band of adhesions at a narrow site of the small intestine. (G) A 10 cm jejunal stenosis (black arrow) with significant proximal intestinal dilation and edema (blue arrow).

One month after her initial consultation, the patient was urgently readmitted with a 5-day history of progressive vomiting and abdominal pain. On clinical examination, the patient presented with abdominal tenderness localized to the periumbilical region with visible bowel patterns. Laboratory analysis indicated severe electrolyte imbalances, including a potassium level of 2.7 mmol/L and a chloride level of 69 mmol/L. The contrast-enhanced CT scan showed a cavernous transformation of the PV. The splenic vein was not visualized, and there was evidence of thrombosis in both the PV and SMV (Fig. 1A). Local small intestinal wall thickened and the lumen narrowed (Fig. 1B). Gastrointestinal radiograph showed localized small bowel stenosis with bird-beak sign (Fig. 1C). Additionally, part of the bowel showed dilation with a gas–liquid level. Enteroscopy was then performed and showed annular stenosis, ulceration, and obstruction of the jejunum approximately 150 cm distal to the pylorus, mucosal congestion and edema throughout the colon, loss of vascular texture and normal structure of the colon (Fig. 1D and E). Due to persistent intestinal obstruction and early enteral nutrition, the patient underwent laparoscopic exploration. Intraoperatively, a 10-cm stenosis of the intestine was identified approximately 140 cm distal to the ligament of Treitz, where there was adhesion between the small intestine, greater omentum, and anterior abdominal wall (Fig. 1F and G). Segmental stricture jejunotomy and double jejunostomy were performed. A small enterostomy reduction was performed 3 months later. Close follow-up was performed and the patient was found to be asymptomatic.

### 3.2. Case 2

**Figure 2.**
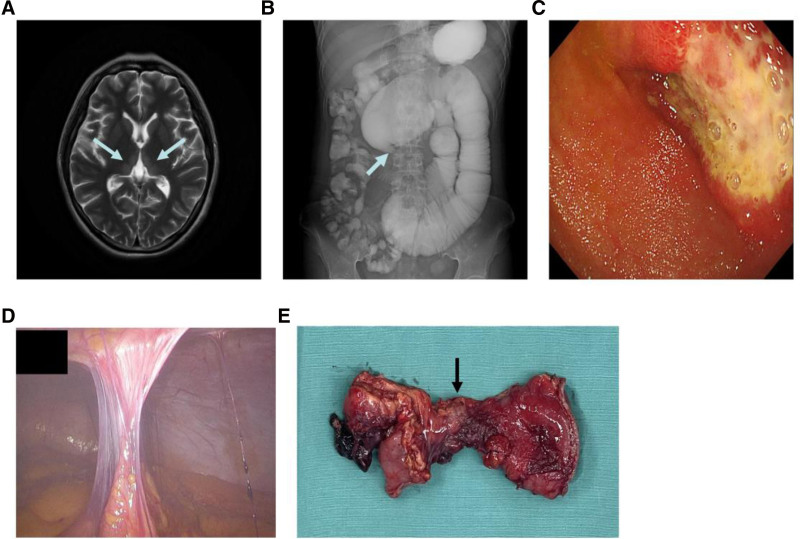
(A) Hyperintense signal shadows are seen near the third ventricle, bilateral thalamus and midbrain ducts (blue arrow), considering Wernicke encephalopathy. (B) Gastrointestinal radiography revealed local stenosis of the jejunum with beak-like changes (blue arrow) and obvious dilatation of the proximal jejunum. (C) Circumferential jejunal stenosis with ulceration. (D) Adhesion was found between mesentery, omentum, and anterior abdominal wall. (E) A 9 cm of the jejunum was resected, the stenotic segment (black arrow) was 2 cm in length.

A 24-year-old female patient was admitted to a local hospital with diffuse abdominal wandering pain. The patient presented with portal thrombosis and subsequently underwent transjugular intrahepatic portosystemic shunt along with PV thrombectomy, followed by the insertion of an indwelling catheter for thrombolysis. Five days after stopping the thrombolytic therapy, her abdominal pain completely resolved, which was confirmed by angiography showing patency of the stent. Anticoagulation therapy was maintained upon discharge (Specific medication regimen not available in local hospitals). Two months later, however, the patient presented with recurrent abdominal pain and hemorrhage, along with symptoms of nausea and vomiting. The abdominal enhanced CT scan showed thrombosis of the PV and SMV, severe stenosis of the proximal PV and cavernous transformation of the PV, which led to admission to our medical facility. Physical examination revealed a distressed facial expression with no apparent tenderness or rebound pain on abdominal palpation. In addition, the patient exhibited rapid eye movements with dizziness and blurred vision, leading to further investigation by cranial magnetic resonance imaging, which confirmed the diagnosis of Wernicke encephalopathy (Fig. 2A). The laboratory findings revealed a severe electrolyte disturbance. One week after anticoagulation therapy (fondaparinux 2.5 mg/day, subcutaneously), the patient’s abdominal pain did not improve significantly. Portal venography showed an absence of blood flow in the stent, with mesenteric venous blood returning to the cavernous transformation vessels of the PV via collateral branches. Imaging showed no flow in the main PV, SMV, or splenic vein. Thrombolytic therapy with an indwelling thrombolysis catheter proved ineffective (50,000 units of urokinase thrice a day was applied for 5 days). Consequently, the patient was transferred to the general surgery department due to worsening abdominal pain. Gastrointestinal radiography identified localized stenosis of the small intestine in group 3, characterized by a beak-like appearance (Fig. 2B), with significant dilation of the proximal small intestine, measuring approximately 10 cm at its widest point. Enteroscopy revealed a small intestinal ulcer with stenosis located 110 cm from the pylorus (Fig. 2C). Surgical intervention was required due to the inability to relieve the obstruction. Intraoperatively, pronounced adhesions were observed in the upper abdomen, presenting as mesh adhesions (Fig. 2D). A portion of the small intestine about 1 m from the ligament of Treitz was completely wrapped by the omentum, and the lumen of this segment was markedly narrow (Fig. 2E). Finally, partial narrow small bowel resection and double enterostomy were performed. Postoperative enteric fluid infusion and nutritional support were performed via a distal enterostomy. Small bowel anastomosis was performed 3 months later.

### 3.3. Case 3

An 18-year-old boy was admitted to a local hospital with persistent fever and altered mental status. He had recurrent abdominal pain and distension with nausea and vomiting for 2 and a half months prior to admission. An enhanced abdominal CT scan showed thrombosis of the PV, SMV, and splenic vein, cavernous transformation of the PV, dilatation of part of the small bowel and edema of its wall. Magnetic resonance imaging of the head revealed Wernicke encephalopathy. Meanwhile, thrombosis was found in the intracranial vein, the right internal jugular vein, and the deep veins of the lower limbs. The etiological screening for thrombosis is considered to be thrombophilia. Portal venography showed no blood flow in the PV, SMV, and splenic vein, leading to the placement of a thrombolysis catheter for treatment. The patient was discharged after improvement and continued oral anticoagulant therapy post-discharge (specific medication regimen not available in local hospitals).

**Figure 3.**
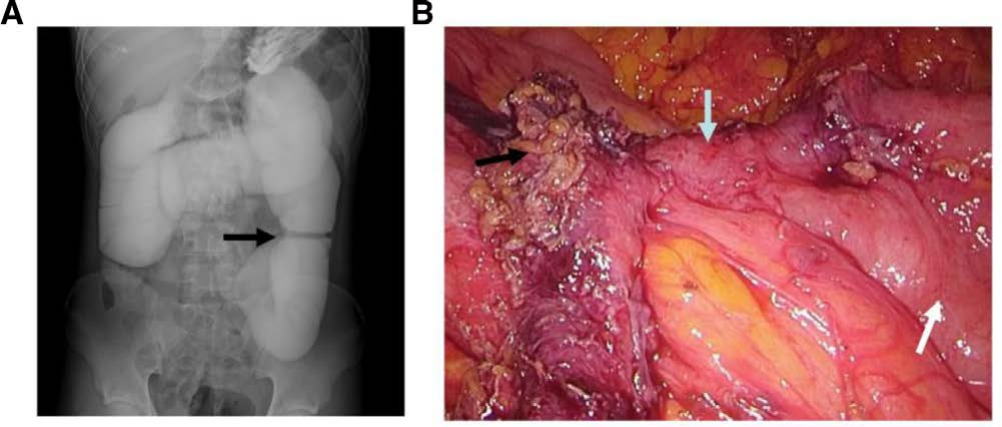
(A) Gastrointestinal radiography showed local stenosis of the small intestine (black arrow). (B) A 6-cm-long stenosis of the jejunum (blue arrow) with significant proximal jejunum dilation (white arrow), and peritoneal adhesion that has been released (black arrow).

Forty days after discharge, the patient presented to our hospital with the same symptoms as before. Laboratory results showed a white blood cell count of 30.60 × 10^9^/L. Gastrointestinal radiography showed a third group of contrast enhancement, indicating small bowel obstruction (Fig. 3A). Subsequently, laparoscopic exploratory surgery was performed, revealing a section of the small intestine about 6 cm long was surrounded by a greater omentum approximately 130 cm distal to the Treitz ligament, and the intestinal cavity was significantly narrowed (Fig. 3B). Segmental jejunotomy and double jejunostomy were then carried out, and the stoma was returned 7 months postoperatively.

### 3.4. Case 4

**Figure 4.**
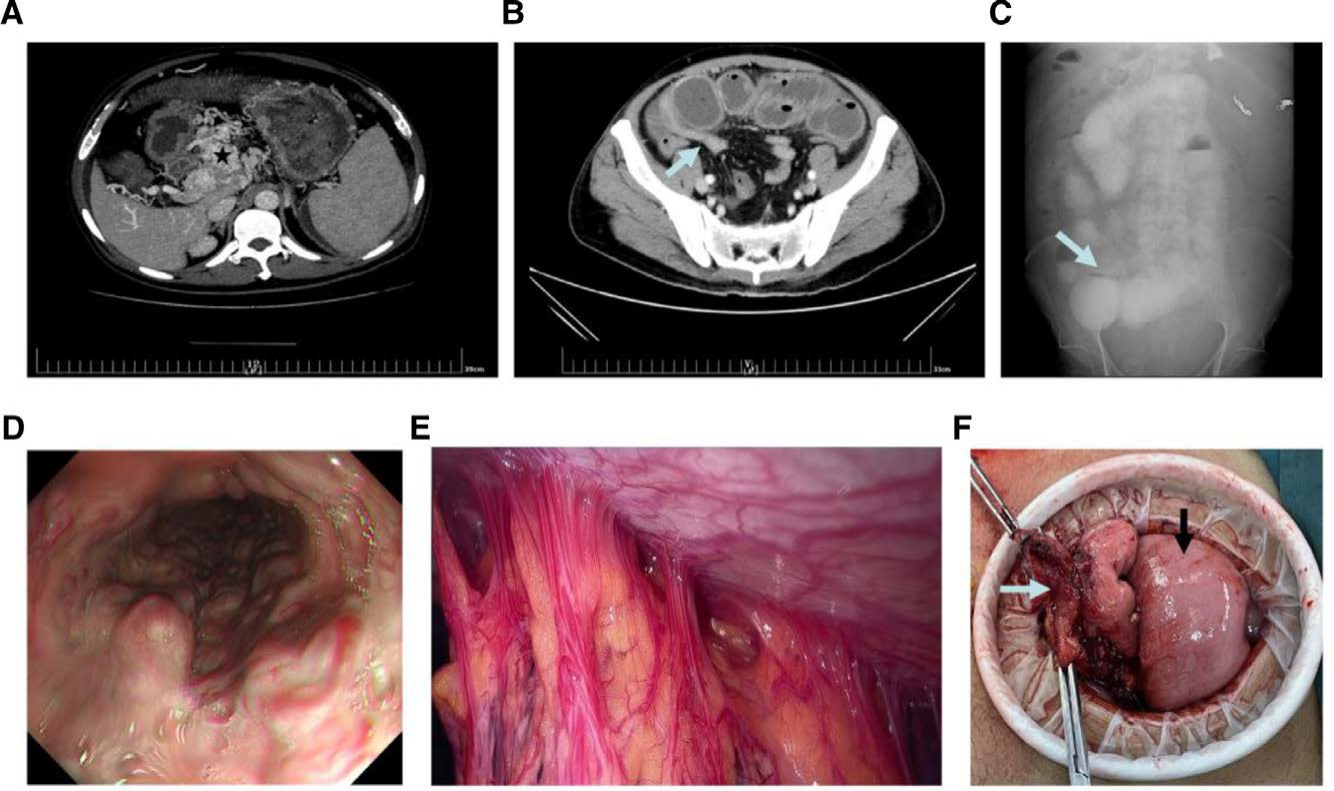
(A) Cavernous transformation of the portal vein, multiple tortuous vascular shadows around the portal vein, around the stomach and the splenic hilum (black pentagram). (B) Local intestinal wall thickened, intestinal lumen narrowed (blue arrow), and proximal small intestine dilated. (C) Gastrointestinal radiography revealed local stenosis of the ileum with a beak sign (blue arrow). (D) Gastroscopy revealed severe esophagogastric varices. (E) Extensive adhesion of bowel, mesentery, and abdominal wall was found during the operation. (F) Narrow section of the ileum (blue arrow), and proximal dilated small intestine (black arrow).

A 50-year-old male patient initially presented to our hospital with a 5-day history of periumbilical pain and nausea. His previous medical history included portal hypertension, splenomegaly, and esophageal and gastric varices for 2 years. Color Doppler sonography revealed old thrombosis of the portal and splenic veins and cavernous degeneration of the PV. These diagnoses were confirmed by an abdominal enhanced CT scan, which also showed the lack of clear visualization of the SMV with the simultaneous formation of multiple peripheral collateral circulation (Fig. 4A). The symptoms on admission were considered to be intestinal ischemic abdominal pain caused by poor intestinal perfusion due to PV thrombosis, which led to immediate initiation of anticoagulation therapy (fondaparinux 2.5 mg/day, subcutaneously). An etiological work-up for portal hypertension and PV thrombosis revealed chronic myeloproliferative disease. The patient was discharged after 10 days without any complaints, while anticoagulation and descending portal pressure therapy were continued after discharge.

The patient was readmitted 4 weeks later for periumbilical pain associated with nausea and vomiting. Abdominal examination revealed tenderness in the upper abdomen without abdominal muscle tension. Laboratory tests revealed hypokalemia, hyponatremia, and hypochloremia. Percutaneous transhepatic portal venography indicated nonvisualization of the left branch of the PV and SMV, with thrombus observed at the confluence of the SMV and splenic vein, and reduced blood flow in the inferior mesenteric vein. A thrombolytic catheter was placed, but the therapeutic effect was poor (40,000 units of urokinase were applied 3 times a day for 7 days). Enhanced CT of the abdomen showed narrowing of the right lower abdominal lumen with local adhesion to the peritoneum and signs of proximal small bowel intestinal obstruction (Fig. 4B). Gastrointestinal radiography showed localized stenosis of the ileum with beak sign (Fig. 4C). Gastroscopy revealed severe esophagogastric varices (Fig. 4D). Emergency surgery was performed in patients with recurrent vomiting and signs of obstruction on imaging. The operation revealed extensive adhesion of the small intestine, greater omentum, and abdominal wall (Fig. 4E), as well as severe dilatation and edema of the small intestine. A 10 cm bowel stricture was identified 2 meters from the ligament of Treitz (Fig. 4F) and a segmental stricture jejunotomy and double jejunostomy were performed. Unfortunately, this patient was lost to follow-up.

## 4. Literature review and discussion

The clinical features and common presentations of intestinal stenosis after PV and SMV thrombosis occurred in the 4 patients in this study were as follows: (1) all patients were initially diagnosed with thrombosis of the portal and SMVs, occasionally accompanied by splenic vein thrombosis. The baseline conditions and coagulation-related indices of the patients on admission are shown in Table 1. All patients had coagulation abnormalities, and a history of ulcerative colitis (case 1), myeloproliferative disorder (cases 2 and 4), and deep vein thrombosis (case 3) were considered as possible risk factors for coagulation abnormalities. (2) All patients were treated with anticoagulation after thrombus detection and coagulation function was subsequently restored (Table 2); (3) the onset of intestinal stenosis in all patients was 4 to 8 weeks after treatment for thrombosis, all the intestinal stenosis was located in the small intestine, with concentric luminal narrowing and ulcer formation; (4) electrolyte disturbances were present in all patients, including 2 patients with Wernicke encephalopathy; (5) different degrees of abdominal adhesions were found during the operation, which may be caused by ischemic inflammatory exudation caused by acute thrombosis.

**Table 1:** Evaluation of risk factors for hypercoagulable states in 4 patients with portal and superior mesenteric vein thrombosis.

Characteristics	Case 1	Case 2	Case 3	Case 4
Age, sex	37, Female	24, Female	18, Male	50, Male
Past medical history	UC	MPD	DVT	PHT, MPD
White blood cells (10^9^/L)	27.06*	9.76	30.60*	12.98
Platelets (10^9^/L)	514*	426*	301*	315*
PT (s)	17.80*	13.32	12.70	12.60
APTT (s)	35.00	36.80	24.90	27.80
TT (s)	15.50	17.80	17.20	14.70
FBG (g/L)	2.85	1.73*	3.10	2.33
FDP (µg/mL)	38.20*	5.40*	7.90*	9.30*
D-Dimer (mg/L)	16.84*	3.28*	2.83*	5.12*
Antithrombin III (%)	92	NA	NA	NA
Protein C (%)	129	54.00*	NA	78
Protein S (%)	42*	14.00*	NA	51*
Homocysteine (µmol/L)	19.80*	6.80	NA	NA
Antinuclear antibodies	Negative	Negative	Negative	Negative
Anticardiolipin antibodies	Normal	Normal	Normal	Normal

DVT = deep vein thrombosis, MPD = myeloproliferative disorder, NA = not available, PHT = portal hypertension, UC = ulcerative colitis.

* Values outside the range of normal.

**Table 2:** Evaluation of coagulation status in 4 patients with portal and superior mesenteric vein thrombosis after anticoagulation therapy.

Items	Case 1	Case 2	Case 3	Case 4
R (min)	12.4*	9.4	5.8	5.2
K (min)	5.5*	2.4	1.8	1.3
α (deg)	40.7*	62	68.3	73.3*
MA (mm)	43.0*	56.6	70.1*	67.2
LY30 (%)	0.0	0.0	0.0	0.9
EPL (%)	0.0	0.0	0.0	0.9
CI	-9.6*	-3.2*	1.5	2.1

α = alpha angle, CI = coagulation index, EPL = estimated percent lysis, K = fibrinogen kinetics time, LY30 = percent lysis 30 minutes after MA, MA = maximum amplitude, R = coagulation factor react time.

* Values outside the range of normal.

We systematically searched the relevant literature in the databases and retrieved a total of 13 papers including 20 patients, the basic information is shown in Table 3. The age of the 20 patients ranged from 24 to 76 years, with a median of 43 years, of whom 14 were male and 4 were female, except for 2 whose sex was not recorded. The proportion of males is significantly higher than that of females, possibly due to the association with venous thrombosis risk. While some previous studies on venous thrombosis have indicated a higher risk in men compared to women, the pathophysiological basis for this phenomenon remains unclear and whether there exists a gender disparity in venous thrombosis risk continues to be a topic of debate.^[16,17]^ Furthermore, our results are in general agreement with data reported in the literature, with the time interval between the presentation of PV and SMV thrombosis and the diagnosis of intestinal stenosis in these 20 patients ranging from 3 to 32 weeks,^[1,4–15]^ with the majority of them occurring at <10 weeks, and 3 older patients at intervals of more than 20 weeks.^[4,11,12]^ Therefore, we recommend that the first follow-up examination should be scheduled about 3 weeks after discharge from the hospital for thrombosis, and regular follow-up every 1 month or so thereafter to ensure early diagnosis and intervention, and that chronic ischemic intestinal stenosis should be considered as one of the differential diagnoses in the presence of abdominal symptoms.

**Table 3:** Literature review of Ischemic intestinal stenosis complicated with portal and superior mesenteric vein thrombosis.

No.	Study	Country	Sex	Age (year)	Length (cm)	Location (cm)	Treatment	Past medical history	Time intervals (week)	Prognosis
1	Eugène C (1995)^[4]^	France	M	36	5	Jejunum (30)	Surgery	antiphospholipid antibody positive	4	Good
			M	46	2	Ileum (20)	Surgery	NA	8	Good
			M	54	2	Jejunum	Surgery	NA	24	Good
2	Antoch G (2000)^[1]^	Germany	M	35	10	Jejunum (100)	Surgery	DVT of the left leg 1 year ago	10	Good
			M	43	20	Jejunum (30)	Surgery	NA	4	Good
3	Narawane NM (2000)^[5]^	India	F	NA	NA	Jejunum	Surgery	Protein C deficiency; factor V Leiden gene mutation	NA	Good
4	Huh SH (2002)^[6]^	Korea	F	40	NA	Jejunum (45)	Surgery	Low antithrombin III History of SMV	12	Good
			M	34	NA	Jejunum	Surgery	NA	3	Good
5	Ho CK (2002)^[7]^	China	F	43	NA	Jejunum	Surgery	Oral contraceptive history of 6 years; Appendectomy 4 years ago	5	Good
6	Kaido T (2004)^[8]^	Japan	F	58	NA	Colon	Surgery	Surgical history of gastric cancer; history of PVT	12	Good
7	Joh JH (2005)^[9]^	Korea	/	/	3	Jejunum (50)	Surgery	NA	6	Good
			/	/	3.5	Jejunum (50)	Surgery	NA	16	Good
8	Yang J (2012)^[10]^	China	M	64	8	Jejunum (50)	Surgery	history of cholecystlithiasis	4	Good
9	Paraskeva P (2015)^[11]^	UK	M	64	NA	NA	Surgery	Pulmonary embolism 14 months previously	28	Good
10	BAO (2020)^[12]^	China	M	67	NA	Ileum (140)	Surgery	Varicose veins of the lower limbs	32	Good
11	Priyadarshi RN (2021)^[13]^	India	M	41	NA	Jejunum (55)	Surgery	Chronic DVT	8	Good
			M	25	NA	Jejunum	Anticoagulant	History of DVT	4	Died
			M	24	12	Jejunum (20)	Surgery	NA	16	Good
12	Bai Y (2022)^[14]^	China	M	50	10	Jejunum (15)	Surgery	Family history of thrombosis	NA	Good
13	Yagi S (2022)^[15]^	Japan	M	76	2	Ileum	Surgery	Chronic myeloproliferative disorder	11	Good

DVT = deep vein thrombosis, F = female, length = length of narrow intestinal segment, location = jejunum/ileum = the distance between intestinal stenosis and the Treitz ligament/ileocecus, M = male, NA = not available, SMV = superior mesenteric vein, time = the time from diagnosis of portal and superior mesenteric vein thrombosis to surgical treatment.

The course of the disease in all included patients can be divided into 2 stages, the clinically characterized by initial acute abdominal pain and fever followed by chronic intestinal obstruction, corresponding to the sequence of thrombosis and intestinal stenosis.^[4]^ The majority of strictures were located in the upper jejunum, with the ileal location being the second most common. Of all the cases included, only 1 was found in both the transverse colon and ascending colon.^[8]^ Consistent with our case studies, 4 cases exhibited jejunal location for intestinal strictures. Unfortunately, there is no precise explanation for why these strictures occur at specific locations; it may be related to thrombus location as well as the number, size, and position of collaterals.^[18]^ Colonic strictures are less commonly reported, which may be due to better collateralization within the colonic venous system.

At present, the occurrence of intestinal stenosis after thrombosis is considered to be a result of chronic ischemia, which is easily misdiagnosed and mistreated, with a very high morbidity and mortality rate, and the mechanism and cause of which are not yet clear. Notably, 1 of our 4 cases had a history of deep vein thrombosis, and of the 20 patients reported, 5 also had a history of deep vein thrombosis or thrombosis.^[1,6,8,13]^ The rest of the history includes long-term oral contraceptives, pulmonary embolism, chronic myeloproliferative disorder, family history of thrombosis, etc. All of these factors may lead to a prethrombotic state, which in turn leads to the formation of microthrombi and increases the risk of developing chronic intestinal ischemia. Therefore, patients who present to the clinic with PV and SMV thrombosis should also be asked about their medical history in detail, and in particular, patients with a previous potentially prothrombotic state should be closely monitored for coagulation, and anticoagulant therapy should be actively given if necessary, and they may also be informed about the subsequent risk of developing intestinal stenosis to increase their vigilance.

There is currently no consensus on the optimal management of PV and SMV thrombosis, which requires a tailored approach based on clinical manifestations, physical examination findings, and imaging results. Treatment strategies typically progress from conservative measures to interventional procedures to surgery.^[19]^ In patients with PV and SMV thrombosis, fasting is recommended to alleviate intestinal vascular dysfunction and edema, while nutritional support therapy is essential to prevent water and electrolyte imbalances. Anticoagulant and thrombolytic therapies play a crucial role in managing acute mesenteric ischemia, as early initiation of anticoagulant therapy can alleviate symptoms and facilitate the development of collateral circulation. The combination of thrombolysis and systemic anticoagulation has been shown to effectively restore blood flow to the ischemic bowel. Surgical intervention becomes imperative upon the onset of peritonitis. Although the etiology of the thrombosis in our cases and those reported in the literature differs dramatically, the clinical consequences are consistent with the manifestation of intestinal stenosis. One patient has been reported in the literature to have received anticoagulation because his underlying conditions were too severe for surgical treatment, and this patient eventually died.^[13]^ Conversely, the remaining patients underwent surgery and had a favorable prognosis. Historically, open surgery has been the conventional approach for intestinal stenosis segment resection and intestinal anastomosis. However, in our study, all 4 patients underwent laparoscopic surgery. Given evident intestinal dilatation and edema, coupled with concerns regarding anastomotic leakage and thrombosis recurrence, the patient underwent resection of intestinal stenosis and a double enterostomy. The technique of postoperative enteric fluid transfusion was used to optimize the nutritional status and maintain the electrolyte balance of the patients. Therefore, the choice and timing of treatment for patients with PV and SMV thrombosis is critical.

## 5. Conclusion

Patients with portal and SMV thrombosis should receive prompt anticoagulant or interventional therapy. Moreover, closer follow-up should be conducted to ensure an earlier diagnosis of ischemic bowel stenosis, which will result in more timely treatment and a better prognosis.

## Acknowledgments

The authors thank all of the patients who participated in the study.

## Author contributions

**Conceptualization:** Zongliang Jia, Zhijian Ren.

**Data curation:** Yuan Chang.

**Formal analysis:** Qian Ma.

**Investigation:** Qian Ma.

**Methodology:** Yuan Chang.

**Project administration:** Zongliang Jia.

**Supervision:** Zhijian Ren.

**Software:** Jie Li, Jun Ke.

**Visualization:** Jun Ke.

**Writing – original draft:** Jie Li.

**Writing – review & editing:** Jie Li, Zhijian Ren, Zongliang Jia.

## References

[1] Antoch G Hansen O Pourhassan S Stock W. Ischaemic jejunal stenosis complicating portal and mesenteric vein thrombosis: a report of two cases. Eur J Gastroenterol Hepatol. 2001;13:707–10. 11434598 10.1097/00042737-200106000-00015

[2] Condat B Valla D. Nonmalignant portal vein thrombosis in adults. Nat Clin Pract Gastroenterol Hepatol. 2006;3:505–15. 16951667 10.1038/ncpgasthep0577

[3] Clavien PA Dürig M Harder F. Venous mesenteric infarction: a particular entity. Br J Surg. 1988;75:252–5. 3349333 10.1002/bjs.1800750322

[4] Eugène C Valla D Wesenfelder L. Small intestinal stricture complicating superior mesenteric vein thrombosis. A study of three cases. Gut. 1995;37:292–5. 7557585 10.1136/gut.37.2.292 PMC1382735

[5] Narawane NM Phadke AY Shah SK Bhandarkar PV Abraham P. Jejunal stricture complicating acute mesenteric venous thrombosis secondary to protein C deficiency and factor V Leiden gene mutation. Indian J Gastroenterol. 2000;19:79–80. 10812821

[6] Huh SH Kim DI Lee BB. Superior mesenteric thrombosis associated with small bowel stricture. Case report. J Cardiovasc Surg (Torino). 2002;43:895–7. 12483187

[7] Ho CK Khoo ST Saw MH. Superior masenteric vein thrombosis. Med J Malaysia. 2002;57:229–32. 24326659

[8] Kaido T Kano M Suzaki S Yanagibashi K Shiota M. Colon stenosis caused by old portal vein thrombosis. Abdom Imaging. 2005;30:358–60. 15654575 10.1007/s00261-004-0247-7

[9] Joh JH Kim DI. Mesenteric and portal vein thrombosis: treated with early initiation of anticoagulation. Eur J Vasc Endovasc Surg. 2005;29:204–8. 15649730 10.1016/j.ejvs.2004.10.005

[10] Yang J Shen L Zheng X Zhu Y Liu Z. Small bowel stricture complicating superior mesenteric vein thrombosis. J Huazhong Univ Sci Technolog Med Sci. 2012;32:146–8. 22282262 10.1007/s11596-012-0026-6

[11] Paraskeva P Akoh JA. Small bowel stricture as a late sequela of superior mesenteric vein thrombosis. Int J Surg Case Rep. 2015;6:118–21. 10.1016/j.ijscr.2014.11.071 PMC4334991 25544479

[12] Haibo BAO Xue SHAO Xiaolu KONG. Primary portal vein and mesenteric vein extensive thrombosis associated with small bowel obstruction in a case. J Gastroenterol Hepatol. 2020;29:284–6.

[13] Priyadarshi RN Anand U Kumar R Thakur SK Bhadani PP Kumar P. Ischemic jejunal stricture in patients with extrahepatic portal vein obstruction. Indian J Gastroenterol. 2021;40:82–7. 33409948 10.1007/s12664-020-01123-x

[14] Bai Y Yongkun Z. A case of jejunal striae atresia due to thrombosis of superior mesenteric vein. J Difficult Dis. 2022;21:644–5.

[15] Yagi S Kioka K Koizumi Y. Ischemic enteritis resulting from polycythemia vera. Clin J Gastroenterol. 2022;15:907–12. 35831680 10.1007/s12328-022-01673-x

[16] Kyrle PA Minar E Bialonczyk C Hirschl M Weltermann A Eichinger S. The risk of recurrent venous thromboembolism in men and women. N Engl J Med. 2004;350:2558–63. 15201412 10.1056/NEJMoa032959

[17] Roach RE Cannegieter SC Lijfering WM. Differential risks in men and women for first and recurrent venous thrombosis: the role of genes and environment. J Thromb Haemost. 2014;12:1593–600. 25081183 10.1111/jth.12678

[18] Ma J Yan Z Luo J Liu Q Wang J Qiu S. Rational classification of portal vein thrombosis and its clinical significance. PLoS One. 2014;9:e112501. 25393320 10.1371/journal.pone.0112501 PMC4231054

[19] Bala M Catena F Kashuk J. Acute mesenteric ischemia: updated guidelines of the World Society of Emergency Surgery. World J Emerg Surg. 2022;17:54. 36261857 10.1186/s13017-022-00443-x PMC9580452

